# The potential of low-intensity and online interventions for depression in low- and middle-income countries

**DOI:** 10.1017/gmh.2016.21

**Published:** 2016-08-19

**Authors:** C. L. H. Bockting, A. D. Williams, K. Carswell, A. E. Grech

**Affiliations:** 1 Department Clinical Psychology, Utrecht University, Utrecht, The Netherlands; 2 Department of Mental Health and Substance Abuse, World Health Organization, Geneva, Switzerland; 3 Department of Health, Mental Health Services, Malta; University of Malta, Msida, Malta

**Keywords:** Depression, internet interventions, interventions, LMICs, mobile health, minimal interventions

## Abstract

The World Health Organization (WHO) reports that low- and middle-income countries (LMICs) are confronted with a serious ‘mental health gap’, indicating an enormous disparity between the number of individuals in need of mental health care and the availability of professionals to provide such care (WHO in 2010). Traditional forms of mental health services (i.e. face-to-face, individualised assessments and interventions) are therefore not feasible. We propose three strategies for addressing this mental health gap: delivery of evidence-based, low-intensity interventions by non-specialists, the use of transdiagnostic treatment protocols, and strategic deployment of technology to facilitate access and uptake. We urge researchers from all over the world to conduct feasibility studies and randomised controlled studies on the effect of low-intensity interventions and technology supported (e.g. online) interventions in LMICs, preferably using an active control condition as comparison, to ensure we disseminate effective treatments in LMICs.

In low- and middle-income countries (LMICs), the majority (76–85%) of people suffering from severe mental disorders receive no treatment at all (World Health Organisation (WHO), 2013). A limited budget for mental health, poor access to services and limited infrastructure, as well as the small number of available mental health professionals contribute to this high non-treatment rate (WHO, 2008; Eaton *et al.* 2011; Patel *et al.* 2011).

The World Health Organization (WHO) reports that LMICs are confronted with a serious ‘mental health gap’, indicating an enormous disparity between the number of individuals in need of mental health care and the availability of professionals to provide such care (WHO, 2010). Traditional forms of mental health services (i.e. face-to-face, individualised assessments and interventions) are therefore not feasible. Given that many LMICs face numerous additional challenges that either preclude large investments in mental health care or hamper the potential benefits that such investments could confer, alternative strategies for addressing this mental health gap are urgently needed.

## Strategies for addressing this mental health gap

We propose three strategies for addressing this mental health gap: delivery of evidence-based, low-intensity interventions by non-specialists, the use of transdiagnostic treatment protocols, and strategic deployment of technology to facilitate access and uptake.

### Delivery of evidence-based, low-intensity interventions by non-specialists

Low-intensity interventions delivered by para-professionals that have been demonstrated to be effective in high-income countries (HICs) might have potential to reduce the gap after adequate adaptation for the local context. There are studies from LMICs demonstrating that psychological interventions, as delivered by non-specialist/lay counsellors, local community health workers (Ali *et al.* 2003; Araya *et al.* 2003; Bolton *et al.* 2014; Chowdhary *et al.* 2015; Patel *et al.* 2010; Rahman *et al.* 2008; Bolton *et al.* 2014; Chowdhary *et al.* 2015) and para-professionals (Bass *et al.* 2006) are effective in reducing depressive symptoms, i.e. for instance in depressed people in India (Chowdhary *et al.* 2015), in depressed pregnant women in Pakistan (Rahman *et al.* 2008) and in depressed adults in Uganda (Bolton *et al.* 2003; Bass *et al.* 2006). Prior to implementation, consideration should be given to several issues around adaptation such as translation of materials to the local language (including appropriate use of expressions or metaphors and literacy-level), cultural differences in belief systems and the perceived appropriateness of different care services and providers (e.g. care delivered in an individual's home or care delivered by an opposite sex service provider), availability of resources to ensure the sustainability of such systems, and legal and ethical frameworks for regulating practice and managing and reporting risk (see Dawson *et al.* 2015). In addition to demonstrating efficacy, research into the implementation of interventions in LMICs (e.g. implementation science) is also required to understand the multiple factors (e.g. implementation approach, health system factors and individual characteristics) that may influence ability to transition effectively to scale (Murray *et al*. 2011).

### Delivery of transdiagnostic treatment protocols

Traditional treatment models primarily adopt a disorder-specific approach (i.e. there are separate protocols for the management of depression and the anxiety disorders). An alternative approach to single diagnosis treatment models is a transdiagnostic approach that can be applied across common mental health problems such as depression and anxiety, as well as the effects of stress and grief. Transdiagnostic interventions address the shared cognitive, emotional and behavioural mechanisms theorised to underpin psychopathology and therefore introduce efficiencies by applying the same treatment principles across different disorders (Barlow *et al.* 2004; McEnvoy *et al.* 2009; Wilamowska *et al*., 2010). First results from trials for transdiagnostic approaches in HICs are promising (Bullis *et al.* 2014; Newby *et al*. 2015). In LMICs transdiagnostic interventions may have wider applicability and greater feasibility for several disorders, including co-morbidity (Murray *et al.* 2014) because they might address a range of common problems using the same manual or techniques, as opposed to multiple manuals for different problems. Low-intensity versions of such interventions may have even greater benefits. A transdiagnostic treatment for symptoms of depression, anxiety and post-traumatic stress delivered by lay workers was studied in Thailand with promising results (Bolton *et al.* 2014). Given the large impact of mental health disorders on the global burden of disease in LMICs (Ferrari *et al.* 2013) and the resources required for scale up of mental health interventions, low-intensity and/or transdiagnostic interventions that reduce the need for multiple intervention protocols may provide a cost-effective solution. However, culturally appropriate unified protocols first need to be developed (with appropriate consideration given to varying diagnostic issues) and then tested in randomised controlled studies in relevant settings.

The WHO is developing and testing a number of low-intensity psychological interventions, including some transdiagnostic versions, with the aim of releasing the manuals for free global use, should the interventions prove efficacious in randomised controlled trials in various LMICs. Two interventions have so far been released, ‘Thinking Healthy’, a manual for the psychological management of perinatal depression (Rahman *et al*. 2008; WHO, 2015) and Problem Management Plus (PM+) (WHO, 2016), which aims to improve management of practical problems and common mental health difficulties that are often associated with these problems (Dawson *et al.* 2015). PM+ is being tested in two randomised controlled trials (Sijbrandij *et al.* 2015, 2016). In addition, WHO and Colombia University plan to release an eight session WHO version of group Interpersonal Therapy for depression in 2016.

Because psychological interventions may face challenges when being scaled up in LMICs, such as the availability of training and supervision, accessibility to interventions and the stigma associated with mental health problems (Patel *et al*. 2011), WHO is also investigating the use of self-help approaches such as a self-help book and pre-recorded audio course (Epping-Jordan *et al*., in press). Self-help may be unguided (e.g. provision of a self-help book) or guided (e.g. provision of a self-help book with support from a para-professional) and has shown good effects in several systematic reviews (Cuijpers & Schuurmans, 2007). Additional research is clearly needed before concluding that initial promising results can generalise to other countries.

### Strategic deployment of technology to facilitate access and uptake

Using technological devices to deliver self-help and guided psychological interventions is likely to be a further alternative and/or additional low-cost strategy to increase the number of individuals that receive treatment in LMICs (see Watts & Andrews, 2014). According to the World Bank (2014), Internet access and the use of technical devices is increasing rapidly in LMICs. According to the International Telecommunication Union (ITU) and UNESCO Broadband Commission for Digital Development report roughly 43% of the total world population has Internet access, with penetration rates as high as 35% in developing countries. Online interventions increase access to mental health care with a minimum of input from a professional, allowing a larger number of individuals to benefit (Andrews & Williams, 2015; Christensen, 2010). Moreover, since online interventions can be accessed from home, these interventions might help in overcoming stigma (Rochlen *et al.* 2004).

Online interventions have been extensively studied in HICs and numerous meta-analyses demonstrate that supported online interventions are effective in treating mental disorders (Andersson & Cuijpers, 2009; Andersson *et al.* 2014; Andrews *et al.* 2010) even when guided by non-specialist support staff (e.g. Titov *et al.* 2010), and when delivered transdiagnostically (see Newby *et al.* 2015 for an extensive review). In LMICs non-specialists/para-professionals could be trained to support these interventions. Adaptations for language, cultural norms and preference for delivery format (e.g. text based *v*. illustrated information) should be taken into account (Chowdhary *et al.* 2014). An online treatment for depression with lay support based on behavioural activation has been developed and the effects will be studied in randomised controlled trials in several LMICs, i.e. Indonesia, China and South Africa (Bockting & Arjadi, 2016: Act and Feel for depression). Furthermore, there are many additional challenges in delivering such interventions in LMICs. These may include limits in confidential access to a device (e.g. if a family share a mobile phone), cost of Internet or mobile use, and ensuring that infrastructure exists for the required maintenance and hosting of online or mobile phone-based interventions (e.g. apps may need to be updated when new operating systems are released). Further, as highlighted by the WHO Mental Health Gap Action Programme (mhGAP), even if local lay counsellors can be trained to support delivery of interventions without loss of treatment fidelity, initial training and/or ongoing supervision may require additional financial and structural resources.

Despite the obvious potential for pragmatic benefits (i.e. low cost, accessibility), few rigorous evaluations of online interventions in LMICs have been conducted. A systematic review of the literature demonstrated that worldwide only three randomised controlled trials of online interventions have been conducted in LMICs for a wide range of mental health problems (i.e. post-traumatic stress disorder, depressive symptoms and internet addiction; Arjadi *et al.* 2015). Therefore we do not currently have sufficient evidence to conclude that supported online interventions are also effective in LMICs (Andersson & Titov, 2014; Arjadi *et al.* 2015).

We therefore urge researchers from all over the world to conduct randomised controlled studies and implementation studies (where an intervention demonstrates efficacy) on the effect of low-intensity interventions and technology supported (e.g. online) interventions in LMICs, preferably using an active control condition as comparison, to ensure we disseminate effective treatments in LMICs (Tol *et al.* 2011). Existing guidelines for establishing the scalability of such interventions should be adopted in this evaluation process (see Tomlinson *et al.* 2013), including cost-effectiveness evaluations that capture all development, infrastructure, and human resource costs. We also encourage standardised reporting of online intervention protocols and outcomes as part of the WHO mHealth Technical Evidence Review Group's mHealth evidence reporting and assessment (mERA) checklist (Agarwal *et al.* 2016). Such endeavours should be supported by rigorous process evaluations that provide an understanding of implementation and problems affecting feasibility. Such qualitative data may help provide guidance for real-world implementation, should the interventions prove efficacious. Hopefully, in this way we can contribute to improve mental health care for those who need it the most in LMICs countries.
